# An Impedimetric Biosensor for Detection of Volatile Organic Compounds in Food

**DOI:** 10.3390/bios13030341

**Published:** 2023-03-04

**Authors:** Alessia Calabrese, Pietro Battistoni, Seniz Ceylan, Luigi Zeni, Alessandro Capo, Antonio Varriale, Sabato D’Auria, Maria Staiano

**Affiliations:** 1 Institute of Food Science, CNR Italy, 83100 Avellino, Italy; 2 URT-ISA, CNR at Department of Biology, University of Naples Federico II, 80126 Napoli, Italy; 3 Department of Engineering, University of Campania Luigi Vanvitelli, 81031 Aversa, Italy; 4 Allinit s.r.l., 84084 Fisciano, Italy; 5 Department of Biology, Agriculture, and Food Science, National Research Council of Italy (CNR-DISBA), 00185 Rome, Italy

**Keywords:** odorant-binding protein (OBP), volatile organic compounds (VOCs), 1-octen-3-ol, trans-2-hexen-1-ol, hexanal, food safety, biosensors

## Abstract

The demand for a wide choice of food that is safe and palatable increases every day. Consumers do not accept off-flavors that have atypical odors resulting from internal deterioration or contamination by substances alien to the food. Odor response depends on the volatile organic compounds (VOCs), and their detection can provide information about food quality. Gas chromatography/mass spectrometry is the most powerful method available for the detection of VOC. However, it is laborious, costly, and requires the presence of a trained operator. To develop a faster analytic tool, we designed a non-Faradaic impedimetric biosensor for monitoring the presence of VOCs involved in food spoilage. The biosensor is based on the use of the pig odorant-binding protein (pOBP) as the molecular recognition element. We evaluated the affinity of pOBP for three different volatile organic compounds (1-octen-3-ol, trans-2-hexen-1-ol, and hexanal) related to food spoilage. We developed an electrochemical biosensor conducting impedimetric measurements in liquid and air samples. The impedance changes allowed us to detect each VOC sample at a minimum concentration of 0.1 μM.

## 1. Introduction

Food safety has substantially impacted human health, the economy, and society [1]. One of the main concerns of the food industry is represented by taints and off-flavors, which may affect consumer food acceptability. Food quality assessment requires the identification of compounds responsible for taints or off-flavors, defined by a broad, heterogeneous group of chemicals named volatile organic compounds (VOCs). The decomposition process that leads to the production of VOCs in food matrices originates from many sources, such as microbiological degradation [2,3,4], contaminations from packaging migration [5,6], contaminated water process, and improper food storage [7], involving the breakdown of carbohydrates [8,9,10], proteins [11,12,13], or lipids [14,15] into their constituents. The oxidation of fats and oils is one of the most predominant phenomena occurring during food life. This chemical process produces VOCs, which decisively influence the sensory quality of foods [16]. Secondary reaction products [17,18,19] (aldehydes, alkanes, alcohols, esters, and epoxides) can continue to react, forming tertiary VOCs such as unsaturated 2-alkenals and 2,4-alkadienals, harmful to human health [20,21,22]. Among the secondary products, hexanal is the most critical indicator for the progress of lipid oxidation [23,24]. Therefore, detecting this rancidity marker can help to characterize the oxidative status of high-fat content food matrices. Furthermore, technological aspects such as the improper use of temperature in the food processing line, packaging conditions, and microbial activity can promote the increase in the concentration of VOCs such as trans-2-hexen-1-ol, described as an astringent and unpleasant odor [25], and 1-octen-3-ol [26] perceived as a moldy, musty, and metallic odor.

Gas chromatography/mass spectrometry (GC/MS) is the methodology of choice for VOC analysis in food. However, despite its proven robustness, it suffers from some drawbacks, such as long-term analysis, time-consuming sample preparation, the need for the presence of a trained operator, and high costs. Hence, novel analytical methods are increasingly required for monitoring food quality in line with the farm-to-fork strategy to ensure consumer confidence. In this scenario, biosensing technologies aim to offer innovative solutions to face the challenges of the food industry.

An example is given by electrochemical biosensors that combine the advantage of specificity with the simplicity of the operation. Furthermore, due to the progress in electronics, these devices can be miniaturized as lab-on-chip for on-site monitoring.

The identification of the biomolecule that specifically recognizes the analyte is the key element in designing a biosensor. Recently, odorant-binding proteins (OBPs) have attracted increasing interest as sensing elements for odors, given their physiological role as odorant carriers to the olfactory neurons. These proteins have been identified in both vertebrates and insects. However, they are structurally unrelated: the first ones belong to the lipocalin superfamily [27,28] and are folded into a β-barrel structure [29], while the second ones show mostly α-helical domains [30]. Furthermore, they are characterized by a compact structure, small size, and extreme thermal and chemical denaturation stabilities [31,32].

In this work, we describe the design of an impedimetric protein-based biosensor for monitoring the presence of three VOCs (hexanal, trans-2-hexen-1-ol, and 1-octen-3-ol) involved in food spoilage using as molecular recognition element (MRE) pOBP. Through direct docking simulation experiments, the binding affinity of pOBP for the selected VOCs was calculated. A competitive assay, based on fluorescence resonance energy transfer (FRET), was performed for each VOC molecule. pOBP was covalently attached to the self-assembled monolayer (SAM) of an α-lipoic acid (ALA)-modified gold surface. The effect of each VOC molecule on the surface electrical properties was monitored by non-Faradaic electrochemical impedance spectroscopy (EIS). The binding phenomena occurring at the electrode interface were observed as a decrease in the imaginary part of impedance with increasing analyte concentrations. The obtained results show the capability of the biosensor to detect the presence of the three selected VOCs in a micromolar range. Therefore, a preliminary study conducted with air contaminated with the selected VOCs shows the maintenance of pOBP capability to recognize the three VOC molecules and suggests that the biosensor could be used as an on-off tool for VOC detection in food.

## 2. Materials and Methods

### 2.1. Materials

Hexanal, trans-2-hexen-1-ol, 1-octen-3-ol, α-lipoic acid, and solvents were purchased from Sigma-Aldrich. Sepharose 4 Fast Flow resin for pOBP purification was acquired from GE Healthcare. DEAE–Sepharose for glutamine-binding protein (GlnBP) purification was obtained from Sigma-Aldrich. Thin-film gold single electrodes (ED-SE1-Au) were purchased from Micrux Technologies (Oviedo, Spain). Materials used for protein electrophoresis were obtained from Bio-Rad. All the used reagents were at analytical or higher grades available. All the solutions were prepared using Milli-Q water.

### 2.2. Direct Docking Analysis

In order to assess the proper binding conformations of the VOC ligands with pOBP, direct docking simulation experiments [33] were performed by MGLTools (http://mgltools.scripps.edu/downloads/ accessed on 8 December 2021), an open-source software suite. After preparing structure files of the protein and the ligands using AutodockTools (ADT) 1.5.6, Autogrid [34] allowed us to calculate the affinity maps through a 3D grid delimiting the ligand-receptor complex. Lastly, with the aid of Autodock, the potential binding poses were predicted, and binding free energies were calculated. The structure files of the ligands were downloaded from PubChem [35] in SDF format and converted into PDB by Open Babel GUI [36]. The structure file of pOBP [37] was obtained by Protein Data Bank [38] (ID: 1E02). The protein crystal structure was processed by removing water and the co-crystallized ligand and by analyzing only chain A. All hydrogen atoms, charges, and atom types were assigned [39,40]. After calculating the ligand torsdof and adding partial charge to atoms, two different PDBQT files were generated. Next, docking simulations were performed considering a grid box of 58 × 66 × 46 points, with a spacing of 0.375 Å. Finally, the affinity maps were computed by Autogrid and saved in a GLG file. The AutoDock Lamarckian genetic algorithm was chosen to perform 100 docking runs, treating the protein as rigid and the ligand as flexible. As a result, 30,000 poses were calculated, and 2,500,000 was set as the maximum energy evaluations. Ultimately, Autodock 4.2 allowed the collection of the docking simulation results in a DLG file consisting of the three-dimensional coordinates of the generated poses. In addition, the binding free energy and the pose clustering were calculated and compared to a reference co-crystallized ligand 1-aminoanthracene. Through AutodockTools, it was possible to identify the amino acid residues involved in binding, exploring the presence of hydrogen bonds or π–π stacking interactions. The lowest binding energy and the higher number of cluster poses were used to identify the best ligand–protein complexes.

### 2.3. Expression and Purification of Recombinant pOBP

The expression and purification of pOBP were performed in accordance with Capo et al. (2018) [41]. In brief, the recombinant pOBP-GST gene, subcloned into expression vector pGEX-2TK, was transformed into the *Escherichia coli* BL21 (DE3) strain. A single *E. coli* colony was picked from an LB agar plate and inoculated overnight in 10 mL LB broth with the selective antibiotic (ampicillin). This starter culture was inoculated in 500 mL of fresh LB medium containing ampicillin (50 μg/mL) for approximately 3 h at 37 °C under shaking. Cells were grown until the absorbance at 600 nm reached a value of roughly 1.0. At this point, the expression of pOBP was induced by adding 0.5 mM isopropyl β-D-1-thiogalactopyranoside (IPTG) for 3 h at 37 °C. The bacterial suspension was centrifuged at 4000 rpm for 30 min at 4 °C. The cellular pellet obtained was resuspended in phosphate buffer saline (PBS) (137 mM NaCl, 2.7 mM KCl, 10 mM Na_2_HPO_4_, 1.8 mM KH_2_PO_4_, pH 7.3) and was incubated at 37 °C for 30 min before with 0.4% Lysozyme, and after with DNase I (50 μg/mL per mL of solution), 5 mM MgCl_2_ (1 mg per mL of solution). Finally, the cells were lysed using an ultrasonic homogenizer (Misonix Sonicator XL Ultrasonic Processor). Centrifugation removed the nucleic acid fragments and cell debris at the end of this step. After filtration, the soluble fraction collected was loaded on a Glutathione Sepharose 4 Fast Flow resin incubated overnight at 4 °C under shaking. After the incubation phase, to remove protein contaminants unbound from the resin, several washes step with PBS were carried out. Then the column was incubated with thrombin (1 unit of thrombin per 100 μg of fusion protein) for 16 h at 25 °C. Finally, after collecting pOBP, the GST tag was removed from the resin with elution buffer (50 mM Tris-HCl, 10 mM reduced glutathione pH 8.0). The purity of the collected protein samples was evaluated by Sodium Dodecyl Sulphate-Poly-Acrylamide Gel Electrophoresis (SDS-PAGE). A single band at about 21 kDa confirmed that the protein was purified to homogeneity. The protein concentration of fractions containing pOBP was estimated by measuring UV absorbance at 280 nm using Jasco V-730 UV/Vis spectrophotometer (molar extinction coefficient 12,200 M^−1^ cm^−1^).

### 2.4. Expression and Purification of Glutamine-Binding Protein

As a negative control of the pOBP-based biosensor, the electrode surface was functionalized with the recombinant glutamine-binding protein (GlnBP). The expression and purification steps were performed following D’Auria et al. (2005) [42]. In brief, *E. coli* cells HB101 expressing GlnBP were grown overnight at 37 °C in LB in the presence of 100 μg/mL ampicillin and then were disrupted by osmotic shock. Next, the crude periplasmic preparations were equilibrated with Na phosphate buffer (pH 7.5) and applied to a DEAE–Sepharose column. Due to the high basicity of GlnBP, there was no adsorption at this pH value, and the protein was collected in the flowthrough fractions. Finally, the protein concentration of GlnBP fractions was estimated by measuring UV absorbance at 280 nm using Jasco V-730 UV/Vis spectrophotometer (molar extinction coefficient 22,920 M^−1^ cm^−1^).

### 2.5. Fluorescence Spectroscopy

Steady-state fluorescence measurements were performed using a Jasco FP-8600 fluorescence spectrometer in a 1.0 cm light path quartz cuvette. Tryptophan (Trp) residues were selectively excited at 295 nm. Emission spectra were acquired in the range of 320–600 nm at 1 nm intervals with a scan speed of 500 nm/min by fixing excitation and emission slit widths at 5 nm. Assays were performed in 500 μL of PBS at pH 7.4 with a volume concentration of ethanol equal to 0.01% [43]. Fluorescence experiments were performed on pOBP samples with optical density lower than 0.1 OD at 295 nm [43,44] to avoid the inner filter effect phenomenon. An ethanolic solution of 1-aminoanthracene (1-AMA) was used as an extrinsic fluorophore after determining its concentration by the Beer–Lambert law using an extinction coefficient equal to 35.45 mM^−1^ cm^−1^ at 280 nm. Titration experiments were performed in triplicate by adding increasing amounts of 1-AMA to pOBP samples. Alcoholic solutions of hexanal, trans-2-hexen-1-ol, and 1-octen-3-ol were tested in competitive binding experiments repeated three times and obtained similar results. Excel 2016 by Microsoft and OriginPro 2021b software were used to analyze the data.

### 2.6. Instruments for Electrochemical Experiments

The impedimetric measurements were carried out with a miniaturized all-in-one electrochemical workstation (MicruX ECStat). The ED-SE1-Au electrochemical sensors are based on a three-electrodes approach: a working, a reference, and an auxiliary electrode.

### 2.7. Surface Derivatization and Functionalization

**Figure 1:**
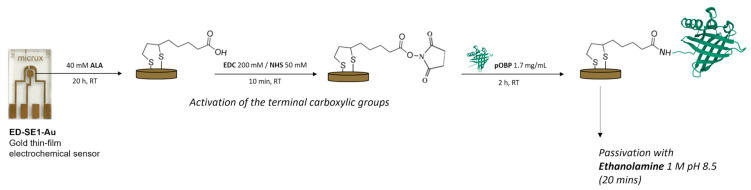
Schematic representation of surface derivatization and functionalization processes.

The gold electrodes were functionalized by slightly modifying the derivatization protocol described by Capo et al. (2022) [45]. Before functionalization, gold electrodes were cleaned by applying 12 potential cycles between −1.0 and +1.3 V with a 0.1 V/s scan rate in 0.05 M sulfuric acid. Then, a self-assembled monolayer (SAM) of thiols was prepared by immersing the clean gold substrates into a 40 mM solution of α-lipoic acid (ALA) for 20 h. Next, the terminal carboxylic groups of the organo-sulfur molecules immobilized on gold electrodes were activated by dropping 15 µL of 200 mM EDC and 50 mM NHS in PBS buffer (pH 7.4) on the surfaces. After 10 min of incubation, the EDC/NHS solution was removed. Subsequently, 15 µL of a 1.7 mg/mL pOBP sample (or GlnBP, used as a negative control) were deposited on the electrodes; after 2 h of incubation, the surfaces were washed three times with sterilized water. Lastly, 10 μL of 1 M ethanolamine (pH 8.5) solution was placed on the surfaces for 20 min to block unreacted active sites. A schematic diagram of the biosensor fabrication is presented in Figure 1.

### 2.8. Impedance Measurements

Non-Faradaic impedance spectroscopy in the absence of a redox probe was chosen to investigate the biorecognition events at the functionalized electrode surface. Binding experiments were carried out at 25 °C and 44% of humidity by depositing 10 μL of hexanal, trans-2-hexen-1-ol, and 1-octen-3-ol dissolved in PBS, preparing serial dilutions, considering the limit of water solubility, at different concentrations (0.1; 0.5; 1; 5, and 10 µM) on the working area of the sensor for 10 min. After rinsing it thoroughly, EIS measurements were conducted in PBS by superimposing a sinusoidal AC potential (0.1 V) to 0 V DC potential in a frequency range of 0.1 to 100,000 Hz. Impedimetric responses are the means of three replicates.

### 2.9. Statistical Analysis

Each impedance measurement was performed in triplicate. The mean and standard deviation were calculated from the value of triplicates. The graphs report the mean values gleaned from the blank values, and the error bars represent the calculated standard deviation. The linear calibration curve was obtained by plotting the change in impedance at 0.1 Hz (ΔZ = Z_baseline_ − Z_VOC_) as a function of the logarithm of the VOC concentration. The limit of detection (LOD = 3.3 σ/S) was determined by considering the slope of the calibration curve, S, and the standard deviation of the response, σ [46]. The graphs were realized in Excel 2016 by Microsoft^®^ and Origin Pro 8.0 software.

## 3. Results and Discussion

**Figure 2:**
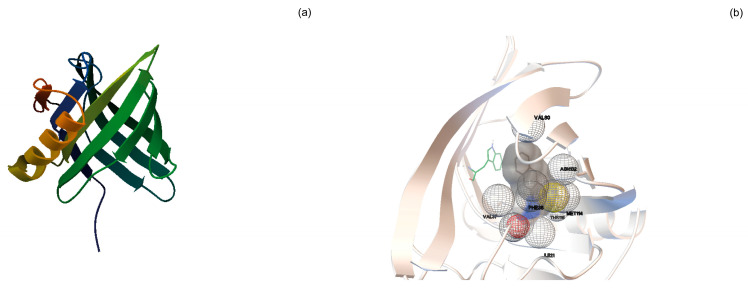
pOBP-1-AMA docking simulation experiments: (**a**) pOBP is a monomer of 157 amino acids containing one disulfide bridge between cysteines at positions 63 and 155 and a single tryptophan residue (Trp) at position 16, accessible to extrinsic fluorophores such as 1-aminoanthracene (1-AMA); (**b**) The image depicts the position of 1-AMA (in gray color) in the β-barrel structure of pOBP. The single tryptophan residue is highlighted in green color.

In this work, intending to develop an electrochemical protein biosensor able to detect the presence of VOCs associated with food spoilage (1-octen-3-ol, hexanal, and trans-2-hexen-1-ol), we selected pOBP as MRE of the biosensor. This protein exhibits a typical hydrophobic cavity that binds VOCs (Figure 2a).

Direct docking experiments were performed to predict binding affinity between pOBP and the selected VOCs. Conformations of pOBP-VOC complexes (Figure 2b) were ranked based on predicted free energy of binding (ΔG), cluster analysis, and ligand position in the binding site. Thus, firstly, the correct position of each ligand in the binding site was checked. Subsequently, the conformation belonging to the most populous cluster, with the lowest estimated free energy of binding, was selected for each ligand. Moreover, it was possible to obtain information on the interactions and the amino acids involved. Finally, docking results were validated with a co-crystallized ligand, 1-aminoanthracene. Table 1 provides the direct docking results sorted in decreasing order according to the estimated free energy of binding (ΔG). Although the ΔG values are quite similar, it is possible to observe that the portion of the alkyl chain and the presence of un-saturations affect the affinity (1-aminoanthracene > 1-octen-3-ol > trans-2-hexen-1-ol > hexanal).

### 3.1. Fluorescence Spectroscopy Measurements

Fluorescence spectroscopy measurements were performed to assess the structural conformation and the binding capacity of pOBP.

In proteins, the indole ring of tryptophan is the dominant fluorophore known to emit fluorescence near 340 nm upon excitation at 295 nm. Monitoring the intrinsic protein tryptophan fluorescence offers the possibility to investigate the relationship structure-function. Any changes in the tryptophan microenvironment may affect the fluorescence emission. A red-shifted fluorescence emission to longer wavelengths may suggest the presence of protein unfolding processes with the loss of protein function [44]. The investigation of protein folding and protein–ligand interactions often relies on the use of the fluorescence resonance energy transfer (FRET) technique. In the case of pOBP, the most widely adopted fluorescence reporter for ligand-binding assays is 1-aminoanthracene (1-AMA). In fact, this fluorescence compound is proven to be a strong ligand for pOBP [47]. One of the emissive characteristics of this compound is to dramatically increase the fluorescence emission when it is bound to a protein matrix.

To develop the FRET assay, the capability of pOBP to bind 1-AMA was evaluated. When excited at 295 nm, 1-AMA showed a weak fluorescence emission with a maximum centered at 537 nm. However, when 1-AMA binds to pOBP, we registered two events: (1) the blue-shift of the emission maximum to 481 nm with the increase in fluorescence emission intensity; (2) the decrease in fluorescence emission intensity at 340 nm. These variations are attributable to the resonance energy transfer phenomenon between the single protein tryptophan residue (at position 16 in the protein structure) and 1-AMA intercalated in pOBP binding site.

**Figure 3:**
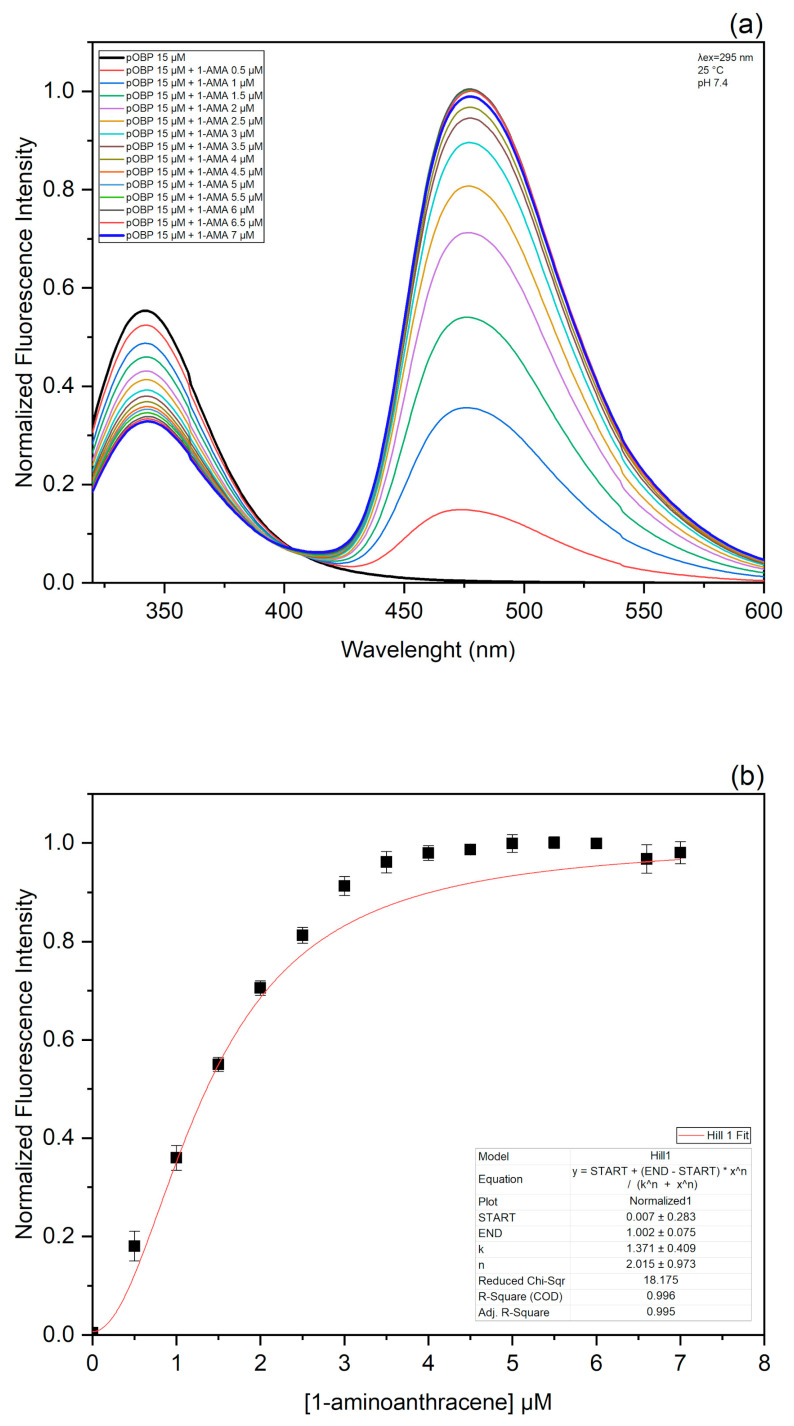
1-AMA titration curve spectra and titration curves fitting: (**a**) The emission spectra of pOBP upon excitation at 295 nm were acquired in the absence and in the presence of different amounts of 1-AMA; (**b**) fluorescence emission intensity at 481 nm was plotted as a function of 1-AMA concentrations.

Figure 3a displays pOBP fluorescence emission intensity increase at 481 nm at increasing 1-AMA concentrations. The analysis of the binding curve indicates the presence of a plateau at 4 mM of 1-AMA, which represents the amount of 1-AMA required to saturate pOBP binding sites (Figure 3b).

#### Competitive FRET Assay

**Figure 4:**
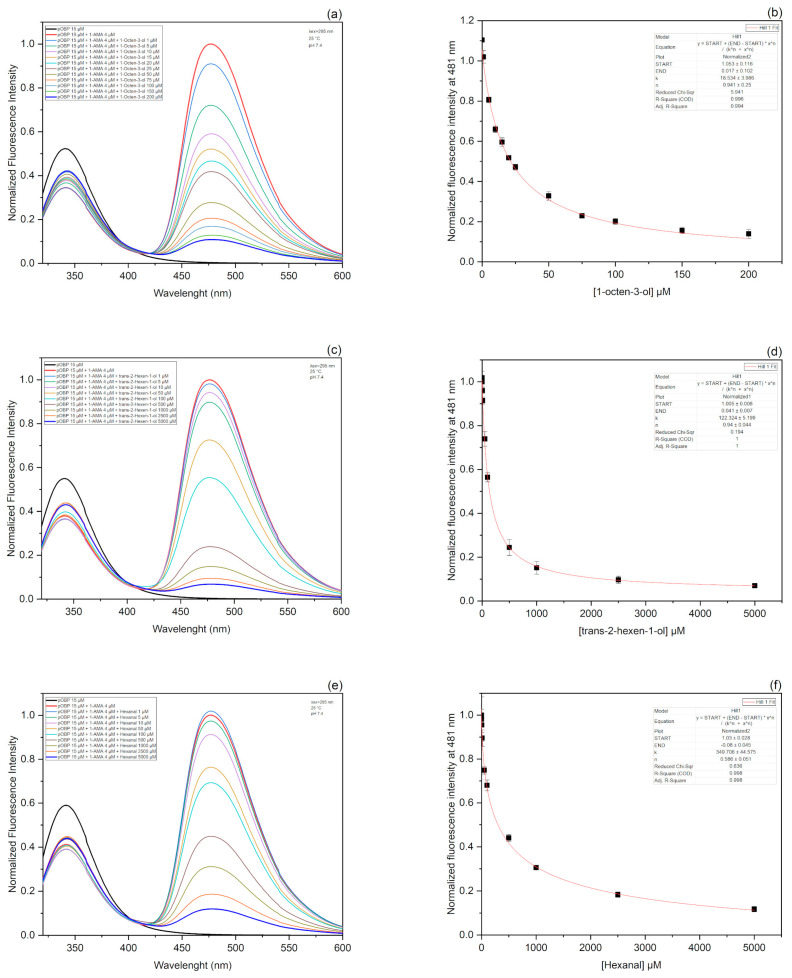
Fluorescence emission spectra and titration curves fitting of 1-octen-3-ol, trans-2-hexen-1-ol, and hexanal. Emission fluorescence spectra of pOBP were acquired in the presence of saturating concentrations of 1-AMA. The addition of increasing amounts of 1-octen-ol (**a**), trans-2-hexen-1-ol (**c**), and hexanal (**e**) determined the decrease in the peak at 481 nm and the increase in the peak at 340 nm. The decreasing intensity of 1-AMA fluorescence emission at 481 nm was plotted as a function of 1-octen-3-ol (**b**), trans-2-hexen-1-ol (**d**), and hexanal (**f**) concentration. The fitting curves obtained by a non-linear function are highlighted in red color.

In order to validate the direct docking results and confirm the protein affinity for the selected VOCs, competitive FRET assays were performed for each VOC molecule. In this assay, the affinity of a ligand was evaluated on its ability to displace the fluorescence probe from the protein complex [48]. More precisely, the competition of 1-octen-3-ol (Figure 4a), trans-2-hexen-1-ol (Figure 4c), and hexanal (Figure 4e) with 1-AMA to the binding site of pOBP were observed by monitoring the displacement of 1-AMA at increasing concentrations of each VOC sample. The titration curves of 1-octen-3-ol, trans-2-hexen-1-ol, and hexanal are presented in Figure 4b,d,f, respectively. The kinetics parameters were calculated by plotting the decrease in the fluorescence intensity at 481 nm as a function of the concentration for each single VOC molecule. The dissociation constant values, calculated through a non-linear fitting function, are 18.5 mM for 1-octen-3-ol, 122.3 mM for trans-2-hexen-1-ol, and 349.7 mM for hexanal.

### 3.2. Functionalization and Immobilization of pOBP on Sensor Surface

pOBP was covalently attached to the self-assembled monolayer (SAM) of an α-lipoic acid (ALA)-modified gold surface. The design of the immobilization layout on the gold surface is crucial for biosensor performance. In fact, it significantly influences the responsiveness of the bio-interface. The electrodes were cleaned by applying potential cycles in the presence of sulfuric acid. Afterward, the gold electrodes were treated as described in detail in “Materials and Methods” (Section 2.7). The surface functionalization was monitored using electrochemical impedance spectroscopy (EIS). Figure S1 (see supplementary material section) shows the Nyquist plot of the gold electrode before and after the immobilization procedure. The results show an increase in the value of the impedance, indicating the presence of covalent immobilization of pOBP on the electrode surface.

### 3.3. Electrochemical Characterization

The electrochemical performance of the biosensor was analyzed by non-Faradaic electrochemical impedance spectroscopy (EIS). This technique allows us to observe binding phenomena at the electrode–electrolyte interface. When a charged electrode surface is placed in an electrolyte, an electrical double layer (EDL) is formed. Its thickness is related to the capacitive variation due to the binding between the target analyte (VOC) and the capture probe (pOBP). A single VOC molecule was dropped from the lowest (0.1 μM) to the highest concentration (10 μM) on the functionalized electrode surface. After incubating for 10 min, the surface was rinsed, and the impedance response was recorded in a PBS buffer.

The same procedure was applied to GlnBP functionalized gold electrodes (the negative control).

The results are presented in the Nyquist diagram, where the imaginary part (Z″ or −Z_img_) is plotted versus the real part (Z′ or Z_real_). The magnitude impedance is a complex number given by:

Z(ω) = Z_real_ (ω) − j Z_img_ (ω), (1)

where ω = 2πf.

**Figure 5:**
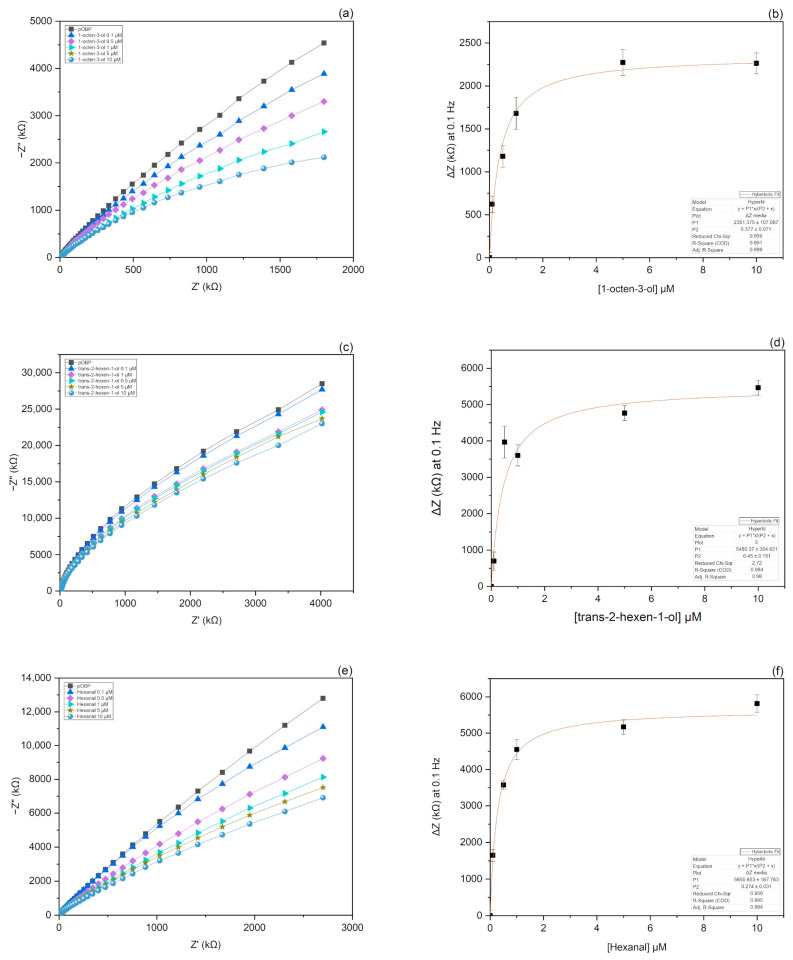
Nyquist plots and binding curves fitting of 1-octen-3-ol, trans-2-hexen-1-ol, and hexanal. Nyquist plots represent the impedance response of the biosensor to increasing concentrations of 1-octen-3-ol (**a**), trans-2-hexen-1-ol (**c**), and hexanal (**e**). As a consequence of the binding events at the interface, the impedance of the electrochemical system increases at increasing concentrations of the VOC samples. Binding curves were obtained by plotting the change in impedance at 0.1 Hz as a function of 1-octen-3-ol (**b**), trans-2-hexen-1-ol (**d**), and hexanal (**f**) concentration. The fitting curves, obtained by a non-linear function, are highlighted in red color.

A non-Faradaic Nyquist plot exhibits a large incomplete semicircle tending to infinity in the low-frequency region. The lack of a redox probe eliminates the parameters associated with electron transfer, such as charge transfer resistance (R_ct_) and Warburg impedance. As a result, the partial semicircle, due to the extremely slow electron transfer, is not followed by the typical diffusion tail [49,50]. Moreover, the solution resistance (R_sol_) in the high-frequency region is attributable to the bulk ionic concentration. Therefore, the impedance of a non-Faradaic sensor is determined by the insulating characteristics of the VOC sample bond to the conductive substrate [51]. The binding phenomena occurring at the electrode interface generate a charge perturbation observable in the Nyquist plot as a decrease in the imaginary part of impedance (−Z″) in the low-frequency regime with increasing VOC concentrations (Figure 5).

The GlnBP-functionalized electrode (negative control) showed no significant impedance variation at 0.1 Hz in the presence of the selected VOC samples in the range of 0.1–10 μM. (Figure S2). These results confirm the specificity of pOBP sensors for the tested VOC molecules.

**Figure 6:**
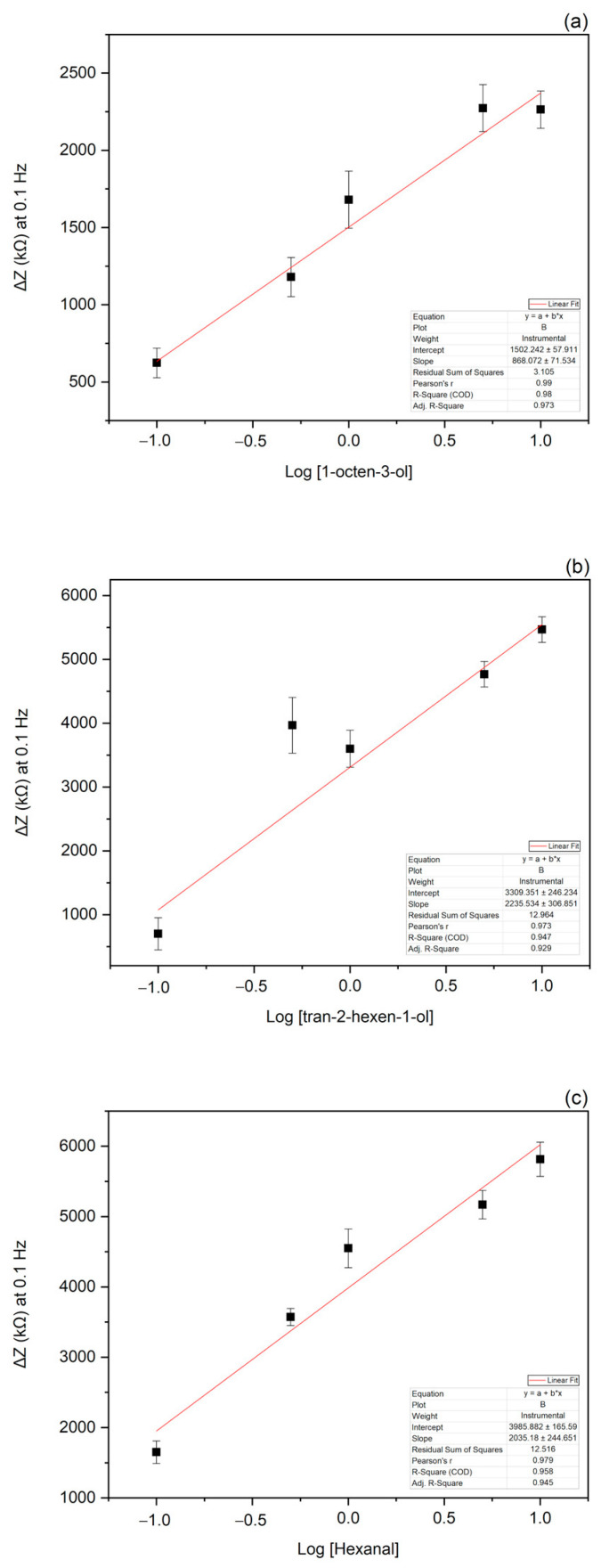
Calibration curves of 1-octen-3-ol, trans-2-hexen-1-ol, and hexanal. The fitting curves were obtained by plotting the logarithmic value of 1-octen-3-ol (**a**), trans-2-hexen-1-ol (**b**), and hexanal (**c**) concentrations versus the change of the impedance value at 0.1 Hz.

Since the maximum signal variation was observed at 0.1 Hz, this frequency was selected to represent the further data. Specifically, the binding curve was obtained by plotting the change in impedance at 0.1 Hz (ΔZ = Z_baseline_ − Z_VOC_) versus the value of VOC concentrations and applying a non-linear fitting function (Figure 5). The limit of detection (LOD) determined as described in “Statistical Analysis” (Section 2.9), was estimated to be 0.49 µM for 1-octen-3-ol, 0.60 µM for hexanal and 0.81 µM for tran-2-hexen-1-ol (Figure 6).

The biosensor showed a response time of 15 min, including the incubation time, and a recovery time of 30 min. For evaluating the long-term stability, the functionalized electrodes were stored in PBS for 20 days at 4 °C with a negligible loss of activity of the biosensor.

### 3.4. EIS Experiments in Gas

pOBP-functionalized gold electrodes were further characterized by electrical impedance spectroscopy (EIS) in air in the absence and in the presence of the selected VOC samples. For this purpose, each electrode was placed inside a suitable gas chamber (Figure S3). Several measurements were acquired until the steady state was reached. Afterward, the gold electrodes were exposed to the single VOC sample, and impedance spectra were collected after 10 and 20 min.

**Figure 7:**
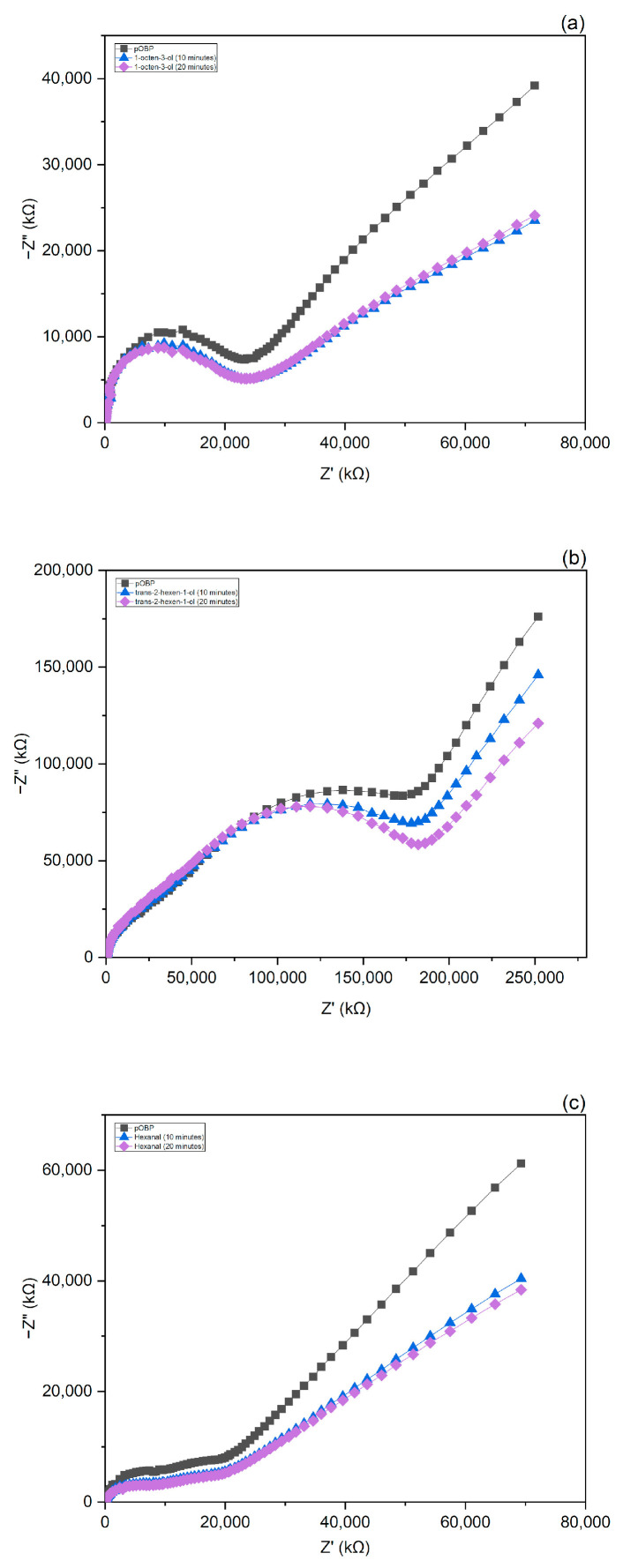
Impedance variations in air as a function of 1-octen-3-ol (**a**), trans-2-hexen-1-ol (**b**), and hexanal (**c**) concentrations.

The Nyquist plots thus obtained (Figure 7) show a variation of the impedance curves in the presence of the volatiles already after 10 min, reaching saturation after 20 min. This result can be ascribed to binding events between pOBP and the VOC samples.

## 4. Conclusions

In this work, we designed a non-Faradaic impedimetric biosensor to detect three different VOC samples (1-octen-3-ol, trans-2-hexen-1-ol, and hexanal) involved in food spoilage by immobilizing pOBP onto a SAM-functionalized gold electrode. The changes in impedance values at 0.1 Hz allowed us to detect the VOC samples in the range of 0.1–10 μM. Furthermore, we observed similar behavior when the biosensor was exposed to air contaminated with the VOC samples. These results suggest that pOBP maintains its functionality outside its living ambient, lending itself to being used as a molecular recognition element even in the gas phase. In the future, to enhance biosensor selectivity, we plan to use the collected data to train neural networks, which could help to discriminate between the different VOC samples. Moreover, by gaining more insight into the type of interactions with the help of molecular docking, we intend to synthesize tailored pOBP mutants with improved selectivity.

## Figures and Tables

**Table 1:** Direct docking simulation results. Ligands are arranged in order of decreasing binding affinity values.

Ligand	ΔG (kcal/mol)	Ki	Amino Acid Residues Involved in Interactions
1-Aminoanthracene	−8.42	674.02 nM	ILE21; PHE35; VAL37; LEU53; VAL80; TYR82; PHE88; ILE100; ASN102; MET114; THR115; GLY116
1-Octen-3-ol	−4.67	379.09 μM	ILE21; PHE35; PHE88; ASN102; THR115; GLY116; LEU118
trans-2-Hexen-1-ol	−3.85	1.50 mM	ILE21; ILE29; PHE35; VAL37; ILE100; SER101; ASN102; MET114; THR115; GLY116
Hexanal	−3.51	2.70 mM	ILE21; PHE35; VAL37; ILE100; SER101; ASN102; MET114; THR115; GLY116

ΔG: Estimated Free Energy of Binding; Ki: Estimated Inhibition Constant; mM: millimolar; µM: micromolar; nM: nanomolar.

## Data Availability

The data presented in this study are available within the article and its Supplementary Materials. Other data that support the findings of this study are available upon request from the corresponding author.

## Supplementary Materials

The following supporting information can be downloaded at: https://www.mdpi.com/article/10.3390/bios13030341/s1, Figure S1: Electrode EIS response before and after the functionalization procedure.; Figure S2: Impedance responses of GlnBP-functionalized electrodes to increasing concentrations of 1-octen-3-ol (a), trans-2-hexen-1-ol (b) and hexanal (c); Figure S3: System used for impedimetric measurements in gas.
