# Supplementary material for: An Impedimetric Biosensor for Detection of Volatile Organic Compounds in Food
Source: Biosensors (Basel). 2023 Mar 4;13(3):341. doi: 10.3390/bios13030341 (PMC10046769; doi:10.3390/bios13030341)
Supplement: Supplementary file 1 [file biosensors-13-00341-s001.zip › biosensors-2230162-supplementary.pdf]

biosensors
MDPI

Supporting Information

An Impedimetric Biosensor for Detection of Volatile Organic Compounds in Food

Alessia Calabrese [1,2,3], Pietro Battistoni [4], Seniz Ceylan [4], Luigi Zeni [3], Alessandro Capo [1,2], Antonio Varriale [1,2], Sabato D'Auria [5,*] and Maria Staiano [1]

1 Institute of Food Science, CNR Italy, 83100 Avellino, Italy
2 URT-ISA, CNR at Department of Biology, University of Naples Federico II, 80126 Napoli, Italy
3 Department of Engineering, University of Campania Luigi Vanvitelli, 81031 Aversa, Italy
4 Allinit s.r.l., 84084 Fisciano, Italy
5 Department of Biology, Agriculture, and Food Science, National Research Council of Italy (CNR-DISBA), 00185 Rome, Italy
* Correspondence: sabato.dauria@cnr.it; Tel.: +39-06-4993-7803

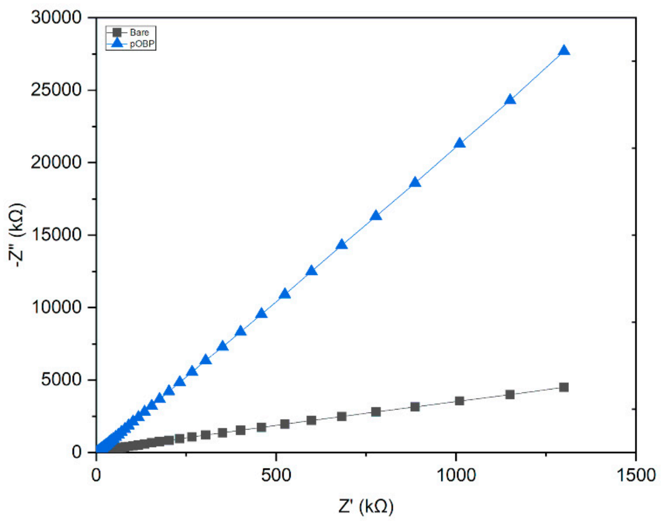

Figure S1. Electrode EIS response before (in gray) and after (in blue) the functionalization procedure. As a consequence of the covalent immobilization of pOBP on the electrode, the impedance of the electrochemical system increases. This variation is ascribable to the increasing thickness of the surface after the assembly procedure.

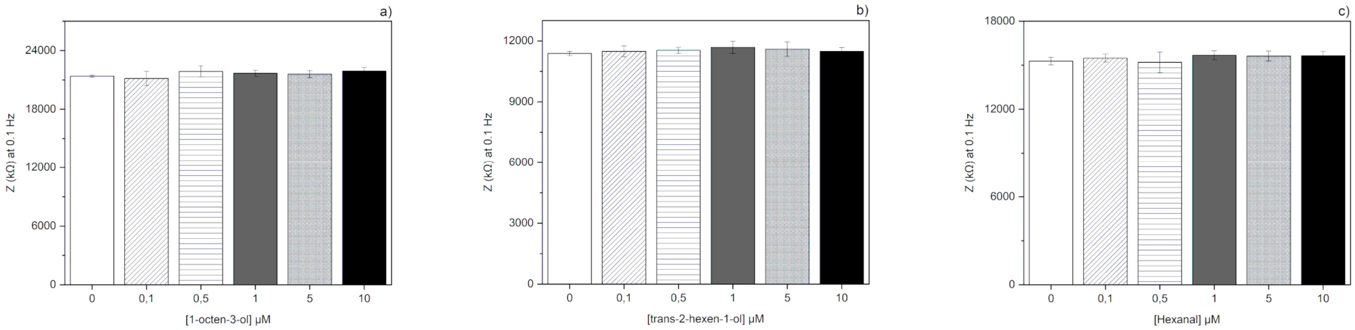

Figure S2. Impedance responses of GlnBP-functionalized electrodes to increasing concentrations of 1-octen-3-ol (**a**), trans-2-hexen-1-ol (**b**) and hexanal (**c**). The GlnBP-functionalized electrode showed no significant impedance variation at 0.1 Hz in the presence of the selected VOCs in the range of 0.1-10 μM.

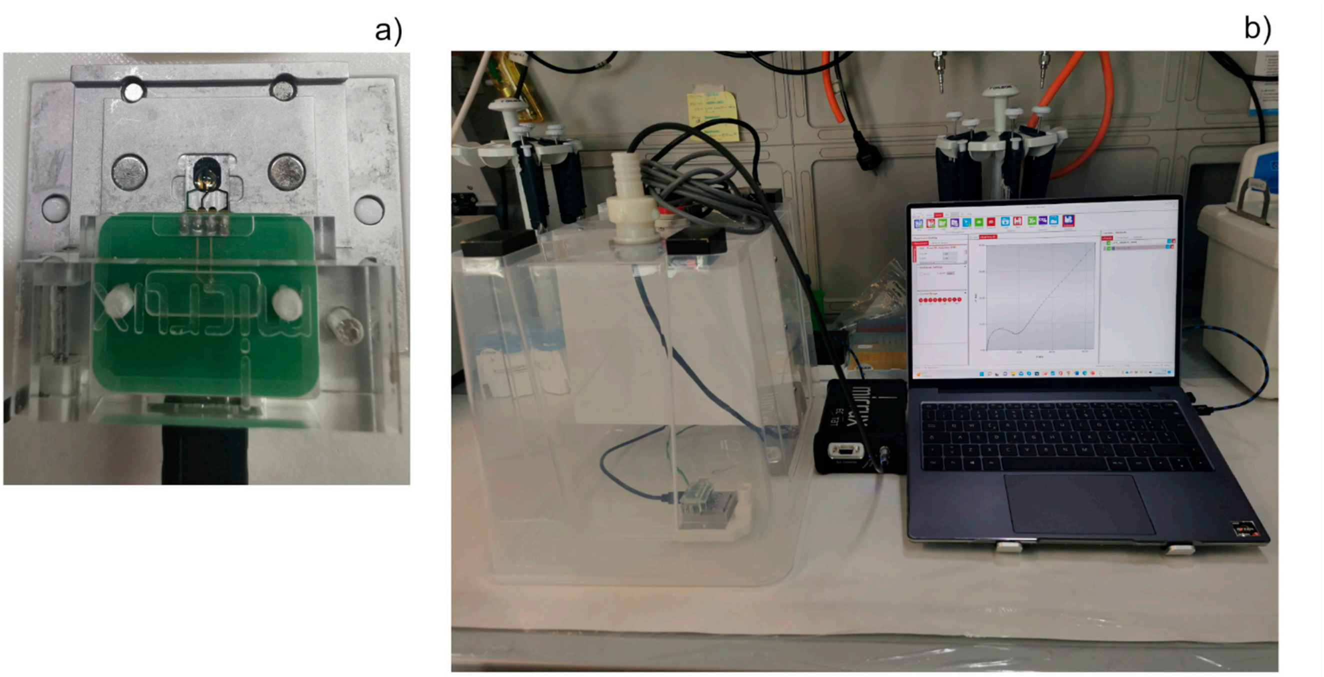

Figure S3. System used for impedimetric measurements in gas. The thin-film gold-based electrode is housed in the electrochemical cell (**a**) and inserted in a saturation chamber. The pure VOC is placed in a Petri dish below the gas chamber. The electrochemical workstation connects the cell to a computer that controls the instrument via the MicruX® EC Manager graphical user interface (**b**).
